# Practical Notes on Cutaneous Subjects

**Published:** 1874-02-01

**Authors:** Tilbury Fox

**Affiliations:** M.D. London, F.R.C.P.


### PRACTICAL NOTES ON CUTANEOUS SUBJECTS.

BY TILBURY FOX, M.D. LONDON, F.R.C.P.

*From the London Lancet, Jan.*, 1874.

ALLEGED VACCINAL SYPHILIS.–I have no hesitation in announcing myself a firm believer in the occurrence of true vaccinal syphilis. I have satisfied myself clinically that it does occur. At the same time, I am quite sure that very many cases to which this designation is applied are in reality only instances of latent heriditary syphilis, excited by the vaccination, and in which the vaccination cannot in any way be fairly blamed for the occurrence of the syphilis. The cases which simulate true vaccinal syphilis are, however, often very successful counterfeits; and a difficulty in disproving that the vaccination itself conveys the syphilitic poison to the attacked, arises oftentimes from the inability to get at any history of syphilis in the parent or parents. The latter naturally try to shift upon vaccination the blame which they should bear themselves. The following case illustrates some of these points in a very admirable manner. It counterfeited vaccinal syphilis; but careful inquiry elicited the fact that the child had no doubt been fully syphilized before it had been vaccinated, and that the vaccination only *excited* the outbreak of the eruption. The father blamed the vaccination, conveniently as it turned out; for no doubt he had infected his wife just after marriage with syphilis. The case is as follows:

Dr.—— brought me a little child, aged one year and two months, with the statement that it had a rash all over it; and that the friends persisted in attributing it to vaccination. The child was vaccinated at three months and a half old; the rash appeared subsequently at the seat of the vaccination, in the first instance, and gradually spread over the body. The rash, suffice it to say, was both papular and tubercular, and consisted of little neoplastic formations of various sizes, some tending to ulcerate and to crust. The child was delicate when I saw it; but it is said to have been well until three months old, when it "had a bad cold, and got out of sorts."

The mother accompanied the child, and on inquiry I learned that this child is the only one she has living; that she has been married five years; that she had a miscarriage when three months gone in the family-way; that an exactly similar mishap was repeated after a while; that then she was prematurely delivered of a still-born child at the seventh month; that this was followed by another miscarriage at three months; and that subsequently the present child was born.

But, further, two months after her marriage she suffered from sores just inside the labia, and was treated for these for some time by caustic, etc. Recently she has lost all her hair, has suffered from violent headaches and deep-seated pains in the bones; the throat has been very sore, and her voice has been altered.

*Remarks.*—At first sight the case might readily have been regarded as one of vaccinal syphilis; but then there was this consideration to be attended to, that the vaccine vesicles healed up in the usual way, and did not themselves become chancrous or abnormally indurated, nor did they ulcerate. The syphilitic rash was at once excited around the inflamed part, and did not behave, *quoad* development, like a secondary rash. But when the history of the mother was inquired into, all doubt as to the source of the syphilization of the child was at an end. The syphilitic poison had been introduced into the mother's system just after marriage, and the child, as it seemed, exhibited its operation upon him just before the date of vaccination, viz.: when the child was three months old, in the commencement of cachexia, and in the presence of "snuffles." The pyrexia attending the vaccinia favored the development of the latent syphilis. The case is of considerable interest in relation to the question of vaccinal syphilis, and is one that might readily be mistaken for the latter by a careless observer. Happily in this case the source of the syphilis was more than usually plainly indicated.

